# Baseline mapping of severe fever with thrombocytopenia syndrome virology, epidemiology and vaccine research and development

**DOI:** 10.1038/s41541-020-00257-5

**Published:** 2020-12-17

**Authors:** Nathen E. Bopp, Jaclyn A. Kaiser, Ashley E. Strother, Alan D. T. Barrett, David W. C. Beasley, Virginia Benassi, Gregg N. Milligan, Marie-Pierre Preziosi, Lisa M. Reece

**Affiliations:** 1 Department of Pathology, University of Texas Medical Branch, Galveston, TX USA; 2 Department of Microbiology and Immunology, University of Texas Medical Branch, Galveston, TX USA; 3 Sealy Institute for Vaccine Sciences, University of Texas Medical Branch, Galveston, TX USA; 4 World Health Organization Collaborating Center for Vaccine Research, Evaluation and Training on Emerging Infectious Diseases, University of Texas Medical Branch, Galveston, TX USA; 5 Office of Regulated Nonclinical Studies, University of Texas Medical Branch, Galveston, TX USA; 6 Initiative for Vaccine Research, World Health Organization, Geneva, Switzerland; 7 Division of Vaccinology, Department of Pediatrics, University of Texas Medical Branch, Galveston, TX USA

**Keywords:** Viral pathogenesis, Vaccines, Viral epidemiology, Viral transmission, Virus-host interactions

## Abstract

Severe fever with thrombocytopenia syndrome virus (SFTSV) is a newly emergent tick-borne bunyavirus first discovered in 2009 in China. SFTSV is a growing public health problem that may become more prominent owing to multiple competent tick-vectors and the expansion of human populations in areas where the vectors are found. Although tick-vectors of SFTSV are found in a wide geographic area, SFTS cases have only been reported from China, South Korea, Vietnam, and Japan. Patients with SFTS often present with high fever, leukopenia, and thrombocytopenia, and in some cases, symptoms can progress to severe outcomes, including hemorrhagic disease. Reported SFTSV case fatality rates range from ~5 to >30% depending on the region surveyed, with more severe disease reported in older individuals. Currently, treatment options for this viral infection remain mostly supportive as there are no licensed vaccines available and research is in the discovery stage. Animal models for SFTSV appear to recapitulate many facets of human disease, although none of the models mirror all clinical manifestations. There are insufficient data available on basic immunologic responses, the immune correlate(s) of protection, and the determinants of severe disease by SFTSV and related viruses. Many aspects of SFTSV virology and epidemiology are not fully understood, including a detailed understanding of the annual numbers of cases and the vertebrate host of the virus, so additional research on this disease is essential towards the development of vaccines and therapeutics.

## Introduction

Severe fever with thrombocytopenia syndrome virus (SFTSV) was first isolated from a patient in China during 2009^1^. DH82 canine macrophage-like cells were infected with the virus and cytopathic effects (CPE), manifested as changes in cell morphology from round to elongated, were observed by light microscopy. After further characterization by electron microscopy and nucleotide sequencing, the virus was shown to be a member of the *Bunyaviridae* family and *Phlebovirus* genus^1^. It is currently classified as a member of the order *Bunyavirales*, family *Phenuiviridae*, genus *Banyangvirus*, and a member of the Bhanja virus serocomplex^2^. Early phylogenetic analysis showed that, although SFTSV had been shown to infect ticks, it was distantly related to viruses from both the *Phlebotomus* fever group as well as the *Uukuneimi* (UUKV) group, indicating that it was part of a new group of phleboviruses^3,4^. As more novel phleboviruses continued to emerge, it was determined that SFTSV is closely related to Malsoor (MV) and Heartland viruses (HRTV), isolated in India and the United States, respectively, and is more distantly related to Palma virus (PALV), originally isolated from ticks in Portugal, and Bhanja virus (BHAV), originally isolated from a goat in India^5–8^. Since its discovery, SFTSV has been isolated from several species of ticks, including *Haemaphysalis longicornis*, *Rhipicephalus microplus*, and *Ixodes nipponensis*, with one study confirming the ability of *H*. *longicornis* to act as a vector for SFTSV^4,9–11^. Based upon recent phylogenetic analyses, it has also been proposed that person-to-person transmission is likely^12,13^.

SFTS was first reported in the Hubei and Henan Provinces of central China in 2009^1,14^. Between 2010 and 2011, it was confirmed that the new disease was caused by a previously unidentified Bunyavirus, now known as SFTSV^3,14,15^. SFTSV infection in humans has an incubation period of ~7–14 days followed by a clinical disease that typically presents as severe fever with thrombocytopenia and leukocytopenia with symptoms persisting ~7–13 days. Patients often also experience lymphadenopathy, gastrointestinal symptoms (nausea, vomiting, diarrhea), central nervous system symptoms (apathy, lethargy, tremors, convulsions, coma), and/or hemorrhagic symptoms (ecchymosis, disseminated intravascular coagulation, pulmonary, and/or gastrointestinal bleeding)^14–16^. In addition, patients often experience multiorgan dysfunction (MOD) in which two or more organ systems are compromised^15–17^. Although damage from MOD may be reversible, it can progress to multiorgan failure (MOF), which is typically associated with fatality in SFTS patients^15–17^. Although MOD is a relatively common outcome of SFTSV infection, there are reports of mild infections that do not require extensive intervention^18^. In addition, there are also reports of unusual disease outcomes, including one study of encephalopathy induced by SFTSV that did not result in a fatality, as well as a report of SFTSV-induced reversible myocardial dysfunction^19,20^. SFTSV infections are most often confirmed using RT-PCR, and although plasma exchange and ribavirin therapy have been used as treatments, their success as therapeutics for SFTS is controversial, as not all treated patients recovered and no results from clinical trials of candidate therapies have been reported to date^14,21,22^. In addition, candidate vaccines are still in the discovery phase.

## Molecular virology and phylogenetics

Like other members of the bunyaviruses, SFTSV has a spherical virion that is covered by an envelope, comprised of a lipid bilayer with glycoprotein spikes, that packages the three RNA segments; large (L) and medium (M) are negative-sense RNA and the small (S) RNA is ambisense^1,23–25^. The L segment, with 6368 nucleotides, encodes the RNA dependent RNA polymerase (RdRp) used by the virus in replication^1,23^. The M segment, 3378 nucleotides, has one open reading frame that encodes a precursor protein that is cleaved in the endoplasmic reticulum to yield two glycoproteins, Gn and Gc^1^, which are embedded in the envelope surface and are involved in virus entry^26,27^. The Gc protein is a class II fusion protein and has been shown to be involved in the fusion of SFTS viral and host membranes^26,28,29^. The Gn protein has been shown to be vital to the incorporation of Gc into the envelope surface and also encodes neutralizing epitopes^27,29^. The S segment is 1746 nucleotides^30,31^ and encodes the nucleocapsid protein (N) and the nonstructural protein (NSs)^1^. Although it has not been confirmed for SFTSV, the glycoproteins of bunyaviruses are suspected of interacting with the N as they have been co-immunoprecipitated with the N in studies using UUKV^32^. The NSs protein is involved in the formation of inclusion bodies that comprise an immune evasion strategy where intracellular vesicles are formed through the interaction of the NSs protein with perilipin A, adipose differentiation-related protein, and synaptogyrin-2^33^. These vesicles sequester TBK1, IKKϵ, and IRF3, which in turn dampens the interferon (IFN) response to the virus^34,35^. These vesicles colocalize with multiple early endosomal markers and autophagy markers, suggesting a role of these pathways in SFTSV pathogenesis^34^. The NSs protein is also involved in inhibition of the IFN response by interfering with the JAK/STAT signaling pathway^36,37^. Deletion of the N-terminal 25 amino acids of NSs negated its ability to suppress the IFN response to the virus^38^.

### Cell culture and virus entry

SFTSV is capable of infecting a variety of cell lines and the DC-specific intracellular molecule 3-grabbing receptor (DC-SIGN) has been shown to be a receptor for SFTSV through interaction with SFTSV glycoproteins, Gc and Gn^39^. The ability of SFTSV to infect cell lines from multiple species, such as hamster, monkey, mouse, and dog, suggests that receptors for the glycoproteins are conserved^39^. DC-SIGNR, liver and lymph node sinusoidal endothelial cell C-type lectin (LSECtin), and glucosylceramide synthase have been shown to also be important for viral entry into cells^39–41^. Gn was shown to bind non-muscle myosin heavy chain IIA (NMMHC-IIA) and overexpression of NMMHC-IIA was found to increase infection in HeLa cells, indicating that NMMHC-IIA is another potential component in SFTSV entry^42^. It was also found that SFTSV infectivity is irreversibly reduced when the pH is lower than 6.0, indicating that pH may influence the conformation of SFTSV glycoproteins^41^.

### Phylogenetic studies

As stated above, SFTSV was found to be most closely related to MV and HRTV, and more distantly related to other phleboviruses, including Rift Valley fever virus (RVFV)^4–6^. The SFTSV RdRp was found to have 46.3% and 73.4% amino-acid similarity with that of RVFV and HRTV, respectively^5,43^. The N of SFTSV and RVFV are only partially homologous, with 41% similarity, whereas the N of HRTV is 62.8% homologous to SFTSV. SFTSV and HRTV glycoproteins share 62.6% sequence homology^43–45^. However, there are ten conserved cysteine residues in the Gn proteins of RVFV, SFTSV, and other related phleboviruses that are involved in the dimerization of Gn^46^. The NSs proteins of SFTSV and HRTV are 63.5% similar while the NSs of SFTSV and RVFV are only 11–16% similar^43^. Maximum likelihood phylogeny for each viral segment further revealed that SFTSV clusters into six genotypes: A, B, C, D, E, and F, whereas earlier analyses also using maximum likelihood divided the strains into five genotypes^31,47–49^. Strains in the F genotype are mostly from China but one analysis included a strain from South Korea^31^. It is also important to note that strains are reported in different genotypes between analyses^4,31^. All genotypes have been found in China, all genotypes but C have been isolated in South Korea, and genotypes B, C, and E have been isolated in Japan^31,50^. It has been shown that the L and M segments of the virus are capable of homologous recombination and/or reassortment in instances of coinfection, thereby causing antigenic shift^48,49,51^. The three segments of the virus have different rates of evolution with the S segment having the highest rate of evolution and the L segment having the lowest^50^.

Owing to the relatively recent discovery of SFTSV, the evolution of the virus has not been fully elucidated. Determination of the phylogenetic history and evolutionary trends of SFTSV would provide information to aid in the development of diagnostics to allow differentiation among related viruses and specific identification of SFTSV in clinical samples.

## Epidemiology

Since its initial discovery, SFTSV has been reported in 23 provinces across China and confirmed human infections were recorded in 18 of these provinces^14^. Several Chinese provinces have had a higher disease burden than others, including Henan, Shandong, Anhui, and Hubei, and cases in these provinces frequently originate from rural, hilly regions^14,52^. Outside of China, SFTSV infections have also been confirmed in Japan, Vietnam, and South Korea, plus evidence in Taiwan and Pakistan. The first reported cases of SFTS in Japan and South Korea occurred in febrile patients hospitalized in 2012 who were confirmed as SFTSV-positive in 2013^10,14,53^. Recently, confirmed cases of SFTSV were reported in Vietnam^54^. Serological surveys in Pakistan suggest that a low percentage of the population have neutralizing antibodies indicative of SFTSV infection and seroconversion^55^. Taiwan has also detected serological evidence of SFTSV as well as viral RNA in some of the surveyed ruminants^56^. As SFTSV is tick-borne, the typical season for SFTSV infection is from early spring through late autumn^14,57,58^ and there are a number of environmental predictors associated with ticks, including cattle density, forest coverage, rainfall, relative humidity, sunshine hours, and temperature elevation^52^. Cumulative data from 2010 to 2016 in China demonstrate that the highest reporting for SFTS cases was between May and July^14^. Reports in South Korea from 2013 to 2015 were highest between July–October, and reports in Japan from 2013 to 2014 were highest between April and August^57,58^. Serological studies throughout China demonstrate that infected individuals undergo seroconversion during the acute and convalescent course of the disease but reports of SFTSV seropositivity amongst healthy populations vary and overall seroprevalence data indicate that only a small portion of the general population has seroconverted (on average ~4.3%)^14,15,59^. Although the age of patients diagnosed has ranged widely, from children to the elderly, adults are more commonly infected and older age is associated with worsening disease outcome^14,58,59^. Specifically, most studies report that the majority of SFTS cases occur in patients older than 50 and case fatality rates (CFRs) are higher in the elderly^14,52,60^.

Reports of CFRs from SFTSV infection range between ~5 and >30% depending on the region surveyed and the amount of reporting in that area^14,57,58^. Owing to the variation in the dates and locations included in published reports of SFTSV incidence, it is unclear how many cases of SFTSV have been diagnosed since its discovery. Between 2011 and 2016, the Chinese Centers for Disease Control and Prevention reported between ~500 and 2500 SFTS cases annually resulting in ~50–100 deaths each year (cumulative CFR from 2010–2016 of 5.3%)^14^. South Korea and Japan tend to report fewer cases and a higher CFR than what is observed in China, however, this may be a result of differences in reporting and surveillance in different regions. South Korea typically reports fewer than 100 cases per year, however, the CFR in South Korea is high (32.6% CFR during 2013–2015)^57^. A retrospective analysis following the first case of SFTS in Japan identified 11 confirmed SFTS patients and six deaths in 2012, and through the end of 2015 a total of 161 cases of SFTS had been reported in Japan^53,58^. Several groups have noted the significant disparity in CFRs between China and other endemic countries. These differences are, in part, attributed to a lack of clinical reporting^61^. Several other factors may also play a role in these disparities.

There have been several isolated, putative infections in Greece, the United Arab Emirates, and the United States between 2009 and 2012, in which SFTSV was considered a possible etiological agent, however, laboratory tests have subsequently indicated that other closely related viruses likely caused these infections^62–64^. In particular, the tick-borne HRTV was identified in the United States in 2012^62^.

A small study comparing fatal and non-fatal cases of SFTS in one region of China identified several differences between clinical symptoms and laboratory biomarkers that correlated with disease outcome^15^. During the first 1–7 days after disease onset, there was no significant difference in viral load, fever, or gastrointestinal symptoms between fatal and non-fatal cases^15^. During days 7–13 post onset, surviving patients had a decrease in viral load, whereas fatal cases continued to have sustained or increased viremia^15^. Both non-fatal and fatal cases were found to develop central nervous system symptoms and hemorrhagic symptoms, but the rate and severity of these manifestations were higher for fatal cases^15^. Both fatal and non-fatal cases experienced apathy, lethargy, and tremors, but convulsions and coma were more strongly correlated with a fatal disease outcome^15^. Similarly, although both fatal and non-fatal cases could manifest MOD, very few surviving patients progressed to the more severe MOF and all fatal cases were associated with MOF. Laboratory tests found that patients who developed fatal MOD/MOF maintained low platelet counts and high levels of the enzymes aspartate aminotransaminase (AST), creatine kinase (CK), creatine kinase-MB (CK-MB), and lactate dehydrogenase (LDH) beyond 13 days post onset, whereas recovering patients typically were experiencing a return to normal platelet and enzyme levels by day 13^15^.

There is ongoing speculation about possible arthropod vectors and reservoir hosts, as studies continue to investigate the transmission cycle of SFTSV. Based on investigation of SFTSV RNA, multiple tick species are implicated as possible vectors while viral RNA has not been detected in mosquitoes, midges, or sandflies^14,65^. *Haemophysalis longicornis* ticks often have a high prevalence compared with other tick populations in China, South Korea, and Japan^14^. In China, in addition to *H. longicornis*, SFTSV RNA has also been detected in *H. concinna* and *Rhipicephalus microplus* ticks^4,65^. In South Korea, SFTSV RNA has been identified in *H. longicornis, H. flava, Ixodes nipponensis*, and *Amblyomma testudinarium* ticks^10,66^. In Japan, multiple tick species have been tested for SFTSV but none have yet been positive for infectious virus or viral RNA^67^. Prevalence rates of SFTSV in tick populations range from 0.6% to 23.5% based on tick species, geographic area, and sampling size^4,10,65,66^. In addition, the various tick species studied are not geographically restricted to the SFTSV endemic regions only. For example, *H. longicornis* is often implicated as a likely vector and can be found outside of China, South Korea, and Japan in countries including Australia, New Zealand, Fiji, New Caledonia, and Russia^68^. Recently, *H. longicornis* was introduced into the United States through agricultural trade and has been found in nine states^69,70^. Although some patients diagnosed with SFTSV report recent tick exposure, not every patient has a known tick-bite history as the apparent infection route^3,14^. Therefore, as regions that are endemic for SFTS do not necessarily have a high prevalence of SFTSV in tick populations, studies are in progress to investigate alternative routes of virus transmission.

As it is likely that ticks acquire SFTSV during feeding on an infected reservoir animal, studies across endemic regions have also tested various animal species for serological markers of infection. Multiple wild and domestic animals surveyed in SFTSV endemic regions of China have been found to carry viral RNA and/or SFTSV-specific antibodies, including sheep, cattle, dogs, pigs, chickens, goats, hedgehogs, geese, and rodents^3,14,65,71–73^. In South Korea, SFTSV RNA and/or antibodies have been detected in Korean water deer, wild boar, feral cats, lizards, and snakes, and in Japan virus-specific antibodies have been detected in cattle and wild boar^66,74–77^. Although SFTSV markers have been identified in several different animal species, there have been only a few reports of disease associated with SFTSV infection. In Japan, disease caused by SFTSV infection has been observed in domestic cats and captive cheetahs^78^. SFTSV-associated disease in a domestic canine has also been reported, with the companion animal suffering from fever, leukopenia, and thrombocytopenia prior to recovery^79^. One group proposed that ticks and migratory birds are responsible for the movement of SFTSV across geographic regions owing to the overlap of bird migratory patterns with the geographic distribution of *H. longicornis* ticks, but the potential role of birds has not been confirmed^72^. Although there is a notable degree of uncertainty about the natural transmission cycle of SFTSV, multiple reports have demonstrated that the virus can be transmitted person-to-person via contact with contaminated blood^13,80^. The majority of patients who are diagnosed with SFTS are farmers who work in fields and possibly have frequent tick exposure, indicating that agricultural work is a major risk factor for SFTS in endemic areas^14,57–59^.

## Diagnostics

### Case definition

Certain laboratory markers, such as elevated AST levels, coupled with relevant patient history, such as time of year, age, and location, can indicate if SFTSV infection is likely; however, the criteria for definitive SFTS diagnosis in China require that SFTSV or viral RNA be detected in patient serum, or patient be positive for anti-SFTSV IgM during active infection, or seroconversion indicated by a fourfold increase in virus-specific IgG in serum drawn during convalescence compared with IgG levels during active infection^81–83^. In Japan, the interim diagnostic criteria for SFTSV infection require a fever >38 °C, gastrointestinal symptoms, leukopenia with <4 × 10^9^ leukocytes/L, thrombocytopenia with <100 × 10^9^ platelets/L, increased lactate, alanine aminotransferase, and AST levels, as well as admission to a hospital owing to symptoms, or death^84^.

### Viral RNA detection

Initially, viral RNA was detected in patient sera during active infection by RT-PCR. In 2012, a quantitative real-time RT-PCR assay was developed with primers targeting the L, M, and S segments that were 99% specific to SFTSV and was capable of diagnosing SFTSV in 98.6% of cases^85^. Another study developed an RT-PCR protocol that was capable of detecting SFTSV, dengue, Hantaan, and Seoul viruses with 99% specificity and 100% diagnosis rate^86^. This two-tube system reduced the cost of running four individual reactions by 40% and provided a quick diagnosis method for multiple viruses with overlapping geographic distributions and similar early clinical presentation^86^. Several other studies have also successfully developed RT-PCR assays to detect SFTSV RNA with all studies achieving a 10 viral RNA copies/μl lower limit of detection^87–89^. Reverse transcription loop-mediated isothermal amplification assays (RT-LAMP) and RT cross-priming amplification (RT-CPA) have also been used to successfully detect SFTSV RNA^90–93^. Multiple reports on the development of RT-LAMP assays to detect SFTSV have shown that those are effective tools for rapid diagnosis with high specificity and sensitivity for SFTSV and the ability to detect multiple strains^90,94^. The RT-CPA assay had a specificity of 94.1% and a sensitivity of 100% compared with real-time RT-PCR and culturing virus from viral samples isolated from 89 suspected SFTSV-infected donors^92^. Both RT-LAMP and RT-CPA assays require ~2 hours to obtain results^90–92^.

### Serological assays

According to the SFTS diagnostic guidelines for China, the disease can be diagnosed by the presence of IgM antibodies in sera during acute illness or IgG antibodies in sera in recovering patients. One study developed an assay for the detection of total antibodies against SFTSV that had no cross-reactivity to hantavirus or dengue samples^93^. The study used a recombinant SFTSV N conjugated to horseradish peroxidase in a double-antigen sandwich enzyme-linked immunosorbent assay (ELISA) for detection of total serum antibodies against SFTSV and was also shown to be effective for testing sera from a variety of species including goats, hedgehogs, pigs, cattle, and chickens with a specificity of 100%^95^. Another study developed an immunochromatographic assay to detect IgG and IgM antibodies by conjugating recombinant SFTSV N (based on strain HB29) and streptavidin with colloidal gold^93^. The Colloidal Gold test was found to be SFTSV specific and as accurate for detecting anti-SFTSV antibodies as indirect immunofluorescence antibody tests previously used for laboratory confirmation of SFTSV infection, with sensitivity to IgG of 1:512 and IgM of 1:128^93^. An indirect ELISA has also been developed that has comparable performance for detection of anti-SFTSV IgG to that of the reference sandwich ELISA kit detailed above, with a specificity and sensitivity of 100%. The same assay can also detect serum IgM with 100% specificity but only 90.59% sensitivity compared with the sandwich ELISA method^96^. Neutralization assays are also a highly regarded method for detecting the presence of SFTSV neutralizing antibodies in serum.

Although great progress has been made with the development of serological and RT-PCR-based diagnostics, there is a lack of standardized reference reagents and a standardized diagnostic methodology that would improve consistency and comparability of surveillance methods and facilitate evaluation of candidate vaccines.

## Immune response

### Innate immune response

In vitro and in vivo analyses have begun to elucidate the innate immune responses resulting from SFTSV infection. Studies in the Golden Syrian hamster model support the importance of IFN signaling in disease prevention as STAT2 knockout hamsters are highly susceptible to SFTSV infection while wild-type hamsters are not^97^. Further highlighting the importance of IFN signaling in protection against SFTSV, in vitro studies have found that SFTSV can antagonize type-I IFN signaling by mechanisms involving virus sequestration of components of the IFN pathway including (but not exclusively) TBK1, IKKε, IRF3, STAT1, and STAT2 into inclusion bodies^35,98^.

Multiple studies have investigated the human immune response to SFTSV infection in an attempt to identify differential profiles of protective and non-protective immune signaling. Specifically, several cohort and case–control studies of SFTS patients in China compared serum levels of cytokines, chemokines, and immune cell subsets with those of healthy individuals, SFTS patients experiencing mild disease, and SFTS patients experiencing severe and/or fatal outcome. Generally, SFTSV infection of humans causes an increase in cytokine signaling that correlates with higher viral load and worsening disease outcome^99–102^. Although there was marked cytokine/chemokine upregulation in all SFTS patients, significant differences in immune signaling between SFTS patients experiencing mild versus severe disease have been identified^99–102^. Specifically, TNF-α, IFN-γ, IP-10, IL-10, IL-6, MIP-1α, IL-8, IL-15, granzyme B, HSP70, G-CSF, IL-1-RA, and MCP-1^99–102^ were present at higher levels in patients with severe compared with mild disease. Not all studies identified statistically significant differences in each of the cytokines/chemokines listed above, however, there is agreement in the literature that severe disease is associated with a “cytokine storm” and increase in pro-inflammatory molecules such as TNF-α, IP-10, and IL-6^99–102^. Several cytokines/chemokines are notably downregulated as a result of SFTSV infection. Specifically, tPAI-1, GRO, PDGF-BB, and RANTES have been detected at decreased expression in SFTS patients when compared with healthy individuals; however, some other studies have not observed any change in RANTES expression based on disease severity^100–102^. One study measured a significant decrease of IFN-β that negatively correlated with disease severity but did not identify similar correlations for IFN-α, IFN-γ, or IFN-λ^99^. The same study found that patients with worsening disease experienced a decrease in IL-1β over time^99^. In addition, this study measured host mRNAs in peripheral monocytes of patients with differing disease severity and found that expression of TLR3, IRF3, and IRF7 was each negatively correlated with disease severity, of which TLR3 exhibited the most downregulation in severe cases^99^.

The role of natural killer (NK) cells in disease outcome is still to be definitively established, as one study found that increased NK cell populations were associated with severe disease, whereas other studies found that NK cells were depleted throughout the first week of symptoms but then began to rise 2 weeks after disease onset^103–105^. One study has investigated the plasmacytoid DC (pDC) and myeloid DC (mDC) populations in patients and determined that levels of circulating pDCs were variable among SFTSV-infected patients but distinct patterns of mDCs could be observed that appeared to be associated with outcome^99^. Specifically, mDCs were significantly increased 1 week after disease onset but then declined in weeks 2 and 3, especially in patients with severe disease^99^. By 3 weeks post disease onset, the surviving patients had significantly more mDCs than deceased patients^99^.

A regulatory role of A20-binding inhibitor of NF-κB activation 2 (ABIN2), and tumor progression locus 2 (TPL2), have been identified in the inhibition of IFN signaling by SFTSV NSs^106^. Direct interactions between NSs and ABIN2 were shown to promote TPL2 complex formation, which subsequently resulted in increased IL-10 expression. Pharmacological inhibition of TPL2 signaling during SFTSV infection resulted in decreased IL-10 expression. Tpl2^−/−^ mice, as well as IL-10^−/−^ mice, survived challenge unlike their wild-type controls. These data show a key role of IL-10 signaling in SFTSV disease severity and outcome.

### Adaptive immune response

Several studies have also measured differences in T- and B-cell subsets in SFTSV-infected humans. Generally, there is agreement in the literature that severe disease is associated with depleted T-cell populations^103–105^. Specifically, patients with severe disease have lower numbers of CD3^+^, CD4^+^, and CD8^+^ T cells throughout the acute phase of disease^103–105^. A study of Chinese SFTS patients reported that arginine deficiency might contribute to T-cell dysregulation during SFTSV infection^107^. In terms of B cells, there are reports that increased numbers of B lymphocytes relative to other types of lymphocytes were correlated with severe disease and fatality^104,105^.

Overall, adaptive immune mechanisms capable of protecting against SFTSV have not been fully identified. Studies with a related bunyavirus, RVFV, have suggested protective roles for neutralizing antibodies^108,109^. Both the nucleoprotein and the Gn/Gc glycoproteins of RVFV have been identified as CD8+ T-cell targets^108,109^ however, the contribution of T cells specific for these proteins to protection against RVFV is incompletely understood.

For SFTSV, there have been a number of studies investigating the induction of virus-specific antibodies, including IgM, IgG, and neutralizing antibodies, resulting from SFTSV infection of humans. A cohort study of 298 Chinese SFTS patients identified some specific patterns of antibody responses following infection that may have relevance to disease pathogenesis^103^. IgM seroconversion occurred between 4 and 21 days after disease onset, peaked after 4 weeks, and decreased significantly thereafter^103^. IgG seroconversion occurred between 2 and 9 weeks post disease onset, peaked at 6 months, and then waned with an estimated half-life of 11 months^103^. During the first 4 weeks of disease onset, low IgM levels were measured in patients who were older, had co-morbidities, had a higher initial viral load, and had severe disease. Moreover, another study of Chinese SFTS patients found that N-specific IgM antibodies were associated with lower viral load and less-severe disease, but N-specific IgG, Gn-specific IgM, and Gc-specific IgM did not have the same correlations^103^. The magnitude and durability of neutralizing antibody responses to SFTSV have not yet been studied in great detail, but a report from China indicates that SFTS patients develop low levels of neutralizing antibodies and although they wane over time, some patients retain neutralizing antibodies through at least 4 years post infection^110^. Of 25 patients tested, 50% plaque reduction neutralization test (PRNT_50_) titers varied between 1:5 and 1:640 during the first year of infection and by year 4 the titers generally decreased only slightly, to a range of 1:20 to 1:160^110^. Based on this 4-year follow-up data, it was predicted that SFTSV neutralizing antibodies could be protective for up to 9 years^111^.

A study in rhesus macaques found that SFTSV infection of non-human primates (NHPs) resulted in increased production of cytokines including IFN-γ and TNF-α similar to that observed in humans^112^. In addition, the study in NHPs found that SFTSV-specific IgM appeared ~5 days post infection (dpi) and declined after ~15 dpi, whereas SFTSV-specific IgG appeared ~7 dpi and plateaued ~15 dpi^112^. Neutralizing antibodies in NHPs were also present at low levels ranging from 1:32 to 1:128. Overall, the immune response in rhesus macaques appeared similar to that of humans but of lower magnitude consistent with the less-severe disease observed in NHPs.

Characterization of humoral responses against other bunyaviruses has indicated that neutralizing antibodies against the viruses typically recognize both Gn and Gc^113^. For SFTSV, neutralizing antibodies to the Gn protein have been identified while non-neutralizing antibodies recognize the N^23,46,114,115^. Using serum samples from four recovered patients as well as SFTSV antisera from mice and rabbits in hemagglutination inhibition (HI) assays, antibodies against SFTSV were found to be cross-reactive with HRTV, with an HI titer of 1:40 to 1:1280, and, to a lesser extent, BHAV, with an HI titer of 1:20 to 1:80^116^. Although the proteins that were bound by antibodies in this study were not specified, cross-reactivity of antibodies against SFTSV with HRTV was corroborated in another study where antibodies to the N from SFTSV bound to HRTV N^117^.

A neutralizing human monoclonal antibody (mAb) for SFTSV was engineered by generating an antibody library from lymphocytes isolated from five individuals infected with SFTSV and was found to bind to the α6 helix in domain III of the Gn protein^46,114^. The mAb was capable of neutralizing several strains of SFTSV, indicating that the epitope is conserved within SFTSV strains and provides a potential vaccine target against SFTSV^46,114^. This mAb was not found to be cross-reactive with RVFV, Forecariah virus (FORV), or PALV^46,115^. In addition, mAbs were derived from a phage display library of lymphocytes from two patients with SFTSV. These monoclonal antibodies bound to the N but had no neutralizing activity^23^. It was determined that the N-terminal region of the N is important for antibody binding as well as three epitopes within the N: Glu10 and Phe11; Lys40, Lys41, and Glu44; and Arg243^23^. Although antibodies that target the N have not been found to have neutralizing activity, they have been demonstrated to be an effective tool for diagnosing SFTSV infection through the recognition of virus in serum^95,96,115^.

### Animal models of SFTSV

Since its discovery, various laboratory animals have been evaluated in an attempt to recapitulate the observed pathogenesis of human SFTSV infection. Table 1 provides an overview of these studies. Small animal models, including mice, rats, and hamsters, have been tested with various pathogenic outcomes. Newborn mice show high susceptibility to SFTSV infection through multiple routes, including intracranial (IC) and intraperitoneal inoculation (IP)^118,119^. Full characterization of newborn infections has yet to be performed though they may provide a lethal model for therapeutics testing. Immunocompetent adult mice generally show few clinical signs of infection^83,97,118,119^, though an in-depth characterization of SFTSV infection in C57BL/6 mice showed similar pathology to mild human infection^120^. Elevated liver enzymes, including AST and ALT, were observed in C57BL/6 mice that were inoculated with 10^5^ TCID_50_ via the IP route and viral RNA was detected in their livers, spleens, and kidneys. To generate a lethal infection in C57BL/6 mice, a dose of the immunosuppressant Mitomycin C was administered^120^. In addition to immune suppression by drugs, immunocompromised knockout mice have also been used to study SFTSV infection. Both C57BL/6 mice deficient in the type-I IFNR (IFNα/βR^−/−^)^83^, and strain 129 mice deficient in type-I IFNR (A129)^119^ have been used for SFTSV model development owing to their immunocompromised status. In both models, lethality was observed with high levels of viral RNA detected in the brain, liver, kidneys and spleen^83,119^. However, these two models show different mean times to death of 3–4 dpi for A129 mice^119^ and 5–7 dpi for IFNα/βR^−^^/−^ mice^83^. STAT1 and STAT2 knockout mice have been utilized with only STAT2 knockouts showing lethal infections^121^. More detail about these mouse models can be found in Table 1.

**Table 1:** Animal models investigated for SFTSV.

Species	Animal strain	Animal ages	Viral strain	Inoculum and route	Lethality	Clinical signs of infection	Ref.
*Mouse*	BALB/C	Newborns	HYSV	3*10^7^ copies IP, IC	L	100% mortality by IC, 35–50% mortality by IP Time to death varies by route and dose.	^118^
	C57BL/6	Newborns	HYSV	3*10^7^ copies IP, IC	L	100% mortality by IC, 35–50% mortality by IP Time to death varies by route and inoculum.	^118^
	CD-1	Newborns	YL-1	10^3^ FFU IC	L	100% mortality after multiple passages in newborn CD-1.	^119^
	Kunming	Newborns	HYSV	3*10^7^ copies IP, IC	L	100% mortality by IC, 40% mortality by IP Necrotic neurons observed when infected IC. Liver damage observed with high viral titers detected in the liver by RT-PCR. Time to death varies by route and dose.	^118^
	IFNAR−/− 129/Sv	Adult	YL-1	10^6^ PFU	L	Virus detected in blood, brain, heart, kidney, liver, lung, and spleen. Antigen detected in brain, heart, intestine, lymph nodes, liver, kidney, and spleen. Time to death 2–3 days.	^119^
	IFNAR−/− C57BL/6	Adult	SD45	10^5^ TCID_50_	L	Necrotizing lymphadenitis in spleen and lymph nodes. Activated macrophages and virus in multiple tissues (spleen, liver, bone marrow). Time to death 5–7 days.	^77^
	BALB/C	Adult	HYSV, HB29	10^5^ TCID_50_ 5*10^7^ IP, IC	NL	Not reported.	^110,112^
	C57BL/6	Adult	HB29 HYSV SD4	10^5^ TCID_50_ IP 5*10^7^ IP, IC	NL	Observed increased megakaryocytes in spleen and bone marrow. Necrosis in the liver. Elevated AST, ALT, and BUN. Reduced WBC. RNA detected in spleen, kidney, and liver. Pre-treatment with Mitomycin C (an immunosuppressant) induces severe/lethal disease within 9–10 days.	^77,110,112^
	CD-1	Adult	YL-1	10^5^ FFU IP	NL	Not reported.	^111^
	Kunming	Adult	HYSV	5*10^7^ copies IP, IC	NL	Not reported.	^110^
	129S1/SvlmJ	Adult	SD4	10^5^ TCID_50_ ID, IP, IM, SC	NL	Not reported.	^77^
	A/J	Adult	SD4	10^5^ TCID_50_ ID, IP, IM, SC	NL	Not reported.	^77^
	BXD34/TyJ	Adult	SD4	10^5^ TCID_50_ ID, IP, IM, SC	NL	Not reported.	^77^
	BXD68/RwwJ	Adult	SD4	10^5^ TCID_50_ ID, IP, IM, SC	NL	Not reported.	^77^
	CAST/EiJ	Adult	SD4	10^5^ TCID_50_ ID, IP, IM, SC	NL	Not reported.	^77^
	DBA/1J	Adult	SD4	10^5^ TCID_50_ ID, IP, IM, SC	NL	Not reported.	^77^
	DBA/2J	Adult	SD4	10^5^ TCID_50_ ID, IP, IM, SC	NL	Not reported.	^77^
	FVB/NJ	Adult	SD4	10^5^ TCID_50_ ID, IP, IM, SC	NL	Not reported.	^77^
	NZBWF1/J	Adult	SD4	10^5^ TCID_50_ ID, IP, IM, SC	NL	Not reported.	^77^
	SLJ/J	Adult	SD4	10^5^ TCID_50_ ID, IP, IM, SC	NL	Not reported.	^77^
	STAT1−/− C57BL/6	6–8 week	YG-1	10 FFU ID	NL	Slight weight loss with recovery. Significant platelet loss without complete recovery by 7 dpi.	^113^
	STAT2−/− C57BL/6	6–8 week	YG-1	10 FFU ID	L	100% lethal by 8 dpi. Significant weight loss as well as platelet loss. Detectable virus in the brain, lungs, liver, spleen, kidney and intestines at 5 dpi.	^113^
	BALB/c	Aged (≥20 months)	CB1/2014	10^6.6^ TCID_50_ IM	NL	Mild weight loss.	^115^
	C3H	Aged (≥20 months)	CB1/2014	10^6.6^ TCID_50_ IM	NL	Mild weight loss.	^115^
	C57BL/6	Aged (≥20 months)	CB1/2014	10^6.6^ TCID_50_ IM	NL	Mild weight loss.	^115^
	FVB	Aged (≥20 months)	CB1/2014	10^6.6^ TCID_50_ IM	NL	Mild weight loss.	^115^
*Hamster*	Syrian Golden Hamster	Newborns	HYSV	5*10^7^ copies IP, IC	L	NR	^110^
	Syrian Golden Hamster *STAT2 KO*	Adult	HB29	1 PFU SC	L	Heterozygous STAT2 mice are resistant to SFTSV infection. Viral RNA detected in serum, liver, spleen, kidney, heart, lung, brain, and intestines. Low levels of platelets observed on day 3. Immature neutrophils elevated between days 2 and 3. Moribund animals had elevated ALP, AST, ALT, GGT, and TBIL. Lesions observed in spleen and liver. Favipiravir provided complete protection against infection.	^90^
	Syrian Golden Hamster	Adult	HB29 HYSV YL-1	10^5^ TCID_50_ IP 5*10^7^ IP, IC 10 PFU SC 10^5^ FFU IP	NL	NR	^90,110–112^
*Rat*	Wistar	Newborn	HYSV	5*10^7^ copies IP, IC	L	100% mortality by IC, 40% mortality by IP.	^110^
	Wistar	Adult	HYSV	1.25*10^8^ copies	NL	NR	^110^
*Ferret*		Young	≤20–24 Months	10^7.6^ TCID_50_	NL	Increase in body temperature at 2 dpi and a return to normal by 12dpi. Slight body weight decrease. Slight thrombocytopenia and leukopenia beginning at 2dpi. Elevated AST and ALT between 2dpi and 8 dpi. Detectable virus in the spleen, liver, kidneys, lungs, and serum with clearance by 8 dpi.	^115^
		Old	≤48–50 Months	10^7.6^ TCID_50_	L	Increase in body temperature at 2 dpi with death occurring by 6dpi. Significant body weight decrease. Severe thrombocytopenia and leukopenia beginning at 2 dpi. Highly elevated AST and ALT by 2 dpi. Detectable virus in the spleen, liver, kidneys, lungs, brain, spinal cord, intestines, and serum.	^115^
*Non-human primates*	Rhesus macaque	4–5 years	HB29	10^7^ TCID_50_	NL	Moderate fever of 4–5 days. Peak viremia of 10^5^ copies/mL. 0.5-fold decrease of WBC. 0.5-fold decrease of platelet count. 0.7-fold decrease of RBC. Elevated ALT, AST, LDH, creatinine, IFN-y, TNF-a, eotaxin, MIP-1B, IL-10, IP-10, IL-5, GM-CSF, and IL-9. Viral RNA detected in spleen, lymph nodes, kidneys, liver (only 1 animal), and intestines. Mild lesions detected in liver and kidneys.	^104^
	Cynomolgus macaque	Not Reported	SD4	10^6^ TCID_50_	NL	Slight drop in platelets in ¼ of animals.	^77^

*L* lethal, *NL* non-lethal, *ID* intradermal, *IV* intravenous, *SC* subcutaneous, *IP* intraperitoneal, *IC* intracranial, *IM* intramuscular, *FFU* focus forming units, *PFU* plaque forming units, *TCID*_*50*_ tissue culture infectious dose.

Rats have also been used to study SFTSV pathogenesis but appear to have limited value as a disease model. In the early characterization of the virus, newborn and adult Wistar rats were used for infections in an effort to recapitulate human disease, however, only newborn rats infected through the IC route showed universal mortality.

Hamsters have previously been employed in the study of other bunyaviruses, such as RVFV, suggesting that they may be a viable model for similar studies with SFTSV^122^. However, much like mouse models, immunocompetent hamsters do not succumb to lethal infections with SFTSV. STAT2 knockout hamsters were found to be susceptible to low doses of virus, succumbing after infection with 10 PFU introduced subcutaneously (SC)^97^.

The use of ferrets as a model has shown significant promise owing to their age-dependent disease manifestations^123^. Young ferrets (<2 years) show little to no clinical manifestations of SFTSV infection. Conversely, aged ferrets (>4 years) show high mortality and notable clinical signs that mirror human infection. Aged ferrets had a 94% mortality rate with severe thrombocytopenia and leukopenia. In addition, AST and ALT levels were increased in comparison with young ferrets^123^. Pathologically, viral antigen was observed in the liver, spleen, kidneys, brain, spinal cord, and intestine. This model shows promise as it is one of the few immunocompetent models to show lethality during infection. This lethality coincides with the observation that SFTSV has significantly more severe manifestations in older individuals suggesting that age is a direct correlate of disease severity^123^.

Infections of NHPs have been performed in an attempt to develop a more biologically relevant model. Two studies have reported the infection of NHPs with SFTSV, one using rhesus macaques^112^ and the other cynomolgus macaques^83^, though only the infection of rhesus macaques was reported in detail. In that study, elevated cytokines mirroring human levels, such as IFNγ^100^, were observed. Minor lesions in the liver and kidney were also observed in the later stages of the infection with viremia peaking at day 5 ~10^5^ TCID_50_/mL and clearing by day 7. Although the rhesus macaques used in that study did become infected, their disease progression mirrored that of only acute SFTSV infection, including mild fever^112^.

To date, no animal model completely recapitulates all the observed clinical manifestations of SFTSV nor has a full characterization of any animal model in particular been performed to date. Consequently, further investigation into suitable animal models needs to be performed in order to best model disease progression and identify models for use in vaccine development.

## Therapeutics and vaccines for SFTSV

### Case management

At present, no approved vaccines or therapeutics are available to prevent or treat infections by SFTSV. Care varies from case to case depending on the patient’s manifestations. Treatment with antivirals and antisera^22^ have shown mixed results. The addition of pulsed steroidal therapy has been shown to have positive effects^124^. To date no standard of care has been established.

### Therapeutics

Early in vitro studies suggested that the common antivirals, ribavirin, and favipiravir, show efficacy in inhibiting viral multiplication. Initially, ribavirin was tested in Vero cell cultures against various strains of SFTSV to determine the 99% effective concentration. Cells were pre-treated with Ribavirin before infection with SFTSV (100 TCID_50_) and cultured for 3 days before viral titers were determined^125^. Effective concentrations were found to range from 19 to 64 mg/ml depending on the strain^125^. However, treatment of cells with similar concentrations of Ribavirin after infection showed drastically diminished effects. From a few reported case studies, the employment of Ribavirin following the diagnosis of SFTSV infection has shown inconclusive effects on disease outcome^21,126^. CFRs and biomarkers of disease, such as viral load and platelet counts, did not show improvement between patients who were given ribavirin and those who were not^21^. These data may suggest that Ribavirin is not a suitable post-exposure treatment, although more studies are required to address its suitability as a prophylactic. However, other case reports in which Ribavirin was administered, indicate positive therapeutic effects and increased patient recovery^21,126^. These contradictory reports show the need for continued investigation into the therapeutic effects of Ribavirin in the treatment of SFTSV infection. Currently, a clinical trial to determine the efficacy of Ribavirin is taking place in China. In addition, two clinical trials are listed in the Chinese Clinical Trial Registry. The first is investigating Ribavirin only (http://www.chictr.org.cn/showprojen.aspx?proj=10477) and the other is to determine the efficacy of Ribavirin in combination with IFN alpha 2a (http://www.chictr.org.cn/showprojen.aspx?proj=11210).

Favipiravir, a purine analog, has also shown effective inhibition of SFTSV replication in vitro as well as in a susceptible animal model^127^. Using IFNα/βR−/− mice, it was shown that Favipiravir provided 100% protection to animals pre-treated with the drug. Animals infected SC with 1.0 × 10^6^ TCID_50_ were dosed via either IP or oral routes with Favipiravir for 5 days. Surviving animals showed milder clinical signs with increased platelet counts and lower viremia, compared with the untreated groups^127^. A clinical trial was performed with Favipiravir to determine its ability to inhibit disease progression in infected patients. Patients were to given a dose of 1800 mg twice on the first day and 800 mg twice a day for the subsequent 9–13 days of treatment^128^. Data from this study are yet to be published but a smaller case study showed promise in the use of Favipiravir as a treatment. Two patients, both male, were given a 5-day course of Favipiravir at different stages of disease^129^. A notable decline in viral load was observed after administration, suggesting a potential effect of Favipiravir on disease course. Further data are required to show statistical significance of these findings.

In addition to these two common antivirals, several other treatments have been proposed for SFTSV infection. The use of recombinant IFN (α, β, and γ) has shown effects in a Vero cell model in a dose-dependent manner^125^. However, IFN administration has yet to be well-characterized in the context of patient infections. A clinical trial has been performed using recombinant human IFN α2, however, those results are not published^130^. Case studies employing l-arginine supplementation have also been performed and suggest that arginine supplementation may be beneficial^107^. In a retrospective clinical investigation, patients treated with calcium channel blockers (CCBs) showed significantly improved clinical outcomes in juxtaposition to patients not receiving CCBs^131^. Animal model testing confirmed that CCBs exhibit anti-SFTSV properties, suggesting that this class of compounds may be a viable option for treatment of patients. Several other molecules have been shown to inhibit SFTSV infection and are shown in Table 2.

**Table 2:** Compounds that have been shown to inhibit SFTSV infection^a^.

Compound	Model	Effective dose	Reference
Ribavirin	Case studies	Case studies show varying results	^21,22,118,124,131^
2’-Fluoro-2’-deoxycytidine	IFNAR−/− mice	3.7 ± 2.0 µM	^132^
Lovastatin	SW13 cells	20 µM	^133^
Fenofibrate	SW13 cells	20 µg/mL	^133^
Caffeic acid	HUH7.5 cells	48 µM	^134^
Amodiaquine	Vero cells	19.1 ± 5.1 µM	^135^
Favipiravir	Cell culture, animal models, and clinical trials	Clinical trials in progress	^120^
Haxachlorophene	Vero and HUH7 cells	IC_50_: 1.3 ± 0.3	^136^
Triclosan	Vero and HUH7 cells	IC_50_: 3.2 ± 0.4	^136^
Regorafenib	Vero and HUH7 cells	IC_50_: 4.5 ± 0.5	^136^
Eltrombopag	Vero and HUH7 cells	IC_50_: 4.1 ± 0.2	^136^
Broxyquinoline	Vero and HUH7 cells	IC_50_: 5.8 ± 1.3	^136^
Arginine	Case studies	Requires further study	^122^
Interferon	Case studies	Requires further study	^121^
N-Butyldeoxynojirimycin-HCL	A549 cells	~50 µM	^137^
Nifedipine	Cell culture, animal models, case studies	Varies in patients	^123^

^a^The mention of specific companies or of certain manufacturers’ products does not imply that they are endorsed or recommended by the World Health Organization in preference to others of a similar nature that are not mentioned. Errors and omissions, except the names of proprietary products, are distinguished by initial capital letters.

Passive transfer of antisera from human survivors has been investigated in small animal models as well as in patients. Sera from survivors were transferred to SFTSV-infected IFNα/βR−/− mice and shown to increase survival and decrease viremia when given 1 hour after infection^132^. Specifically, sera containing neutralizing antibodies at a titer of 1:2000 were given once every 24 hours to mice infected with 10^6^ pfu of the virus.

Several case reports have described the use of serum transfer as a treatment for SFTSV infection. One study found that the administration of serum from survivors to patients with rapidly progressing SFTSV infection had a positive effect on disease outcome. All patients treated with serum transfusion showed lower viremia levels as detected by RT-PCR and 13 of the 14 patients treated with serum transfusion survived^132^. These data suggest that serum transfer may be a viable treatment for patients with rapidly progressing SFTSV infection, but the mechanism of protection requires further study, although it is assumed to be neutralizing antibodies.

Overall, Favipiravir, IFN, and antiserum treatment have the potential to be possible therapies for SFTSV infection. Nonetheless, continued research into viable treatments is required and the development of new therapeutics is needed.

### Vaccines

Much like therapeutics, SFTSV vaccine development has been limited and there are no licensed vaccines currently available. Multiple studies, utilizing various approaches, have shown varying levels of efficacy in preventing SFTSV-associated disease in animal models. Recombinant protein strategies^133^, DNA vaccine strategies^134–136^, and viral vector strategies^137^ have all been utilized and several of these studies are discussed below.

The first study evaluated recombinant SFTSV NSs (100ng) given in Freund’s adjuvant (which is not an acceptable adjuvant for humans) and was administered twice (14 days apart) to C57BL/6 mice, via the SC route. Mice were challenged with 3 × 10^7^ pfu SFTSV via the IP route. Despite high titers of anti-NSs antibodies, no inhibition of viral replication nor accelerated viral clearance were observed in vaccinated mice^133^. The second study investigated a DNA vaccine candidate. Plasmids encoding the NSs and N genes were transfected into mice and immune responses were measured. Significant differences in the release of TNF-α from CD8+ and CD4+ cells were observed in mice transfected with NSs in comparison to N, and both groups of vaccinated animals had increased immunologic response compared with controls. Protective efficacy of this strategy cannot be assessed as a subsequent challenge with the virus was not reported^134^.

Two other DNA vaccine studies have shown the promise of this strategy. Kwak et al.^135^ developed multiple constructs that varied in their expression of each of the five SFTSV proteins. The glycoproteins of SFTSV were found to confer the highest protection when used in the lethal ferret model, with partial protection induced by the NSs, N, and RdRp vaccines^135^. Researchers found that doses as low as 40 µg of plasmid induced a strong SFTSV-specific response with robust protection against intramuscular challenges as high as 10^7.6^ TCID_50_. All of the candidate vaccines induced robust T-cell responses and strong, antigen specific antibodies were developed.

Studies by Kang et al. showed that the inclusion of an IL-12 open reading frame in addition to SFTSV genes helped to induce strong cellular responses^136^. Owing to the stability and ease of production, as well as the efficacy observed in these studies, the use of a DNA-based vaccine may be a promising approach to address the need for a SFTSV vaccine.

Of the candidate vaccines reported to date, the use of the viral vector strategy appears to be the most promising. Two studies have demonstrated the effectiveness of viral vectors; one employing a recombinant vesicular stomatitis virus (rVSV) system and another utilizing a recombinant, rationally attenuated SFTSV. The rVSV system encodes the glycoproteins of SFTSV and was given as a single 1 × 10^4^ pfu dose to IFNα/βR^−/−^ mice^137^. Immunization with this recombinant VSV vaccine was performed via multiple routes including IP, IV, SC, and intranasal. All immunized animals were protected from an IP challenge of 2 × 10^4^ pfu of the Wuhan strain of SFTSV^137^. In comparison with the unvaccinated control group that all succumbed to SFTSV infection, the immunized animals showed minimal clinical signs of infection.

The study of the rationally attenuated recombinant SFTSV demonstrated that two variants, a NSs knockout and a single amino-acid mutant can convey protection in a lethal ferret model^138^. Both variants inhibited viral replication after post vaccination challenge and decreased most clinical signs. High genetic stability of the NSs knockout virus was observed even after six passages, demonstrating a low likelihood of reversion^138^.

Work has been undertaken to investigate candidate strains of SFTSV on which to base future vaccines so as to maximize protective coverage. Strain HB29 was found to be cross neutralized by rabbit antibodies generated against eight other SFTSV strains, and with sera from 33 SFTS survivors, suggesting that it is broadly neutralized and may be an effective base for candidate vaccines^139^. Other groups have taken these studies further and begun to map the antigenic regions of SFTSV’s glycoproteins, including identifying subdomain III of Gn as a target for neutralizing antibodies^46^. Determining antigenic regions that also induce neutralizing antibodies may be key in the development of certain types of vaccines.

Overall, the fields of SFTSV therapeutics and vaccines are in early stages and a significant gap in knowledge remains. Determining the correlate(s) of protection, molecular mechanisms of immune subversion, and the development and characterization of effective therapeutics are crucial to combatting SFTSV infection.

## Conclusions

At present, no licensed vaccines are available for SFTS, and research is in the discovery stage with only three published reports of vaccine candidates. Existing drugs are available that may be efficacious in the treatment of SFTSV infection, though more preclinical data and clinical studies are required. In addition, no specific vector control methodologies have been effective at curbing the expansion of SFTSV competent vectors. Animal models for SFTS appear to recapitulate many facets of mild and/or severe human disease, however, none of the models mirrors all clinical manifestations. There are major gaps in knowledge with insufficient data available on basic immunologic responses, the correlates of protection, and the determinants of severe disease by SFTSV and related viruses.
